# Upon the Mode of Action of a Group of Poisons

**Published:** 1869-04-01

**Authors:** L. Herman

**Affiliations:** From La Gazette Médicale, and the Archiv. Fur Anatomie, Physiologie und Wissenschaftliche Medicin


### *Upon the Mode of Action of a Group of Poisons.*

BY L. HERMAN, FROM LA GAZETTE MÉDICALE, AND THE ARCHIV. FUR ANATOMIE, PHYSIOLOGIE UND WISSENSCHAFTLICHE MEDICIN.

The author has studied the group of anæsthetics of which ether and chloroform are the best known; but this group comprises many other bodies, amylene, clorethyl, and its derivative chlorides; acetic ether, and many other combinations; the ethylic, methylic and amylic alcohols; and lastly three gases, protoxyd of nitrogen, chloride of methyl and ethylene.

All these bodies have, in his opinion, the common property of dissolving the sanguineous globules in the plasma, as Böttcher and Von Wittich have demonstrated for ether and chloroform.

With reference to volatile substances there is no doubt of it, but as to gases, it has not been demonstrated, except of chloride of methyl.

How do these substances act upon the nervous system? Does the destruction of the globules act upon the nervous system, either through the medium of the respiration, deranged in its principal condition, the introduction of oxygen, or by the action of the hæmato-globuline set free. But this is not probable, for several reasons.

1st.—There may be intense anæsthesia without extensive destruction of globules. For the destruction of globules of the blood in circulation there is a very sensitive test, the presence of biliary coloring matter in the urine; now icterus although common in "*chloroformization*" is far from being a constant phenomenon.

2d—The respiration cannot be the cause of the phenomena observed, first, because they are not dyspnœic in their nature, and next because anæsthetics act upon the frog, an animal almost insensible to respiratory lesions.

3d.—Free hæmato-globuline cannot be the cause of these phenomena, for anæsthetics act upon invertebrata with absolutely colorless blood, as well as upon red-blooded vertebrata.

According to M. Herman there exists in the nerve-substance and in the blood corpuscles an identical matter, *protagon*, upon which anæsthetics act.

Liebreich has demonstrated the existence of protagon in the brain, and L. Hermann has demonstrated it in the globules. This matter is dissolved by the anæsthetics, sufficient quantities of which may be received into the blood. The gaseous anæsthetics, on the contrary, and especially the chloride of methyl, are not dissolved in sufficiently large quantities in the blood, and hence only cause the blood globules to swell, without effecting their solution. As to the two other gases, they are dissolved too scantily in the blood to permit the detection of any appreciable action upon the red-globules.

This interpretation leaves a desideratum. How does it happen that bodies, without appreciable action upon the *protagon* of the globules, cannot act upon the protagon of the nervous substance with sufficient effect to produce the phenomena of anæsthesia?

The author explains it by the delicacy of the material processes of the cerebral substance, an explanation which, he admits, leaves still a desideratum.

It is asked, next, if *all* bodies dissolving the sanguineous globules might not have likewise this action upon the nervous centres, and in investigating, in this point of view, the biliary acids, he has seen his supposition confirmed. He cites in illustration the nervous phenomena of severe icterus.

M. Hermann concludes his memoir by an appendix, in which he gives the process of extracting the *protagon* of the blood and indicates its characteristics.

